# Binucleation ramps up gene expression meeting the physiological demands of an organism

**DOI:** 10.1371/journal.pbio.3001639

**Published:** 2022-05-25

**Authors:** Ari S. Dehn, Vicki P. Losick

**Affiliations:** Boston College, Chestnut Hill, Massachusetts, United States of America

## Abstract

This Primer explores the implications of a new PLOS Biology study that shows how polyploidy, via binucleation, enables Caenorhabditis elegans intestinal cells to ramp up gene expression, supplying oocytes with the necessary lipids for optimal organismal growth and reproductive fitness.

## Teaser

In this issue of *PLOS Biology*, van Rijnberk and colleagues show how polyploidy, via binucleation, enables *Caenorhabditis elegans* intestinal cells to ramp up gene expression supplying the oocytes with the necessary lipids for optimal organismal growth and reproductive fitness.

Polyploidy results from the duplication (or more) of a cell’s entire diploid genome and is recognized as a conserved and widespread mechanism for cell growth in development, tissue repair, and disease [1]. Polyploid cells are common in many organisms, including yeast, nematodes, plants, insects, fish, and mammals [2]. However, polyploid cells can exhibit distinct ploidy states in terms of both their nuclear ploidy and number of nuclei per cell. A key gap in our understanding is how a cell’s ploidy state impacts cellular, tissue, organ, and organism physiology.

**Fig 1:**
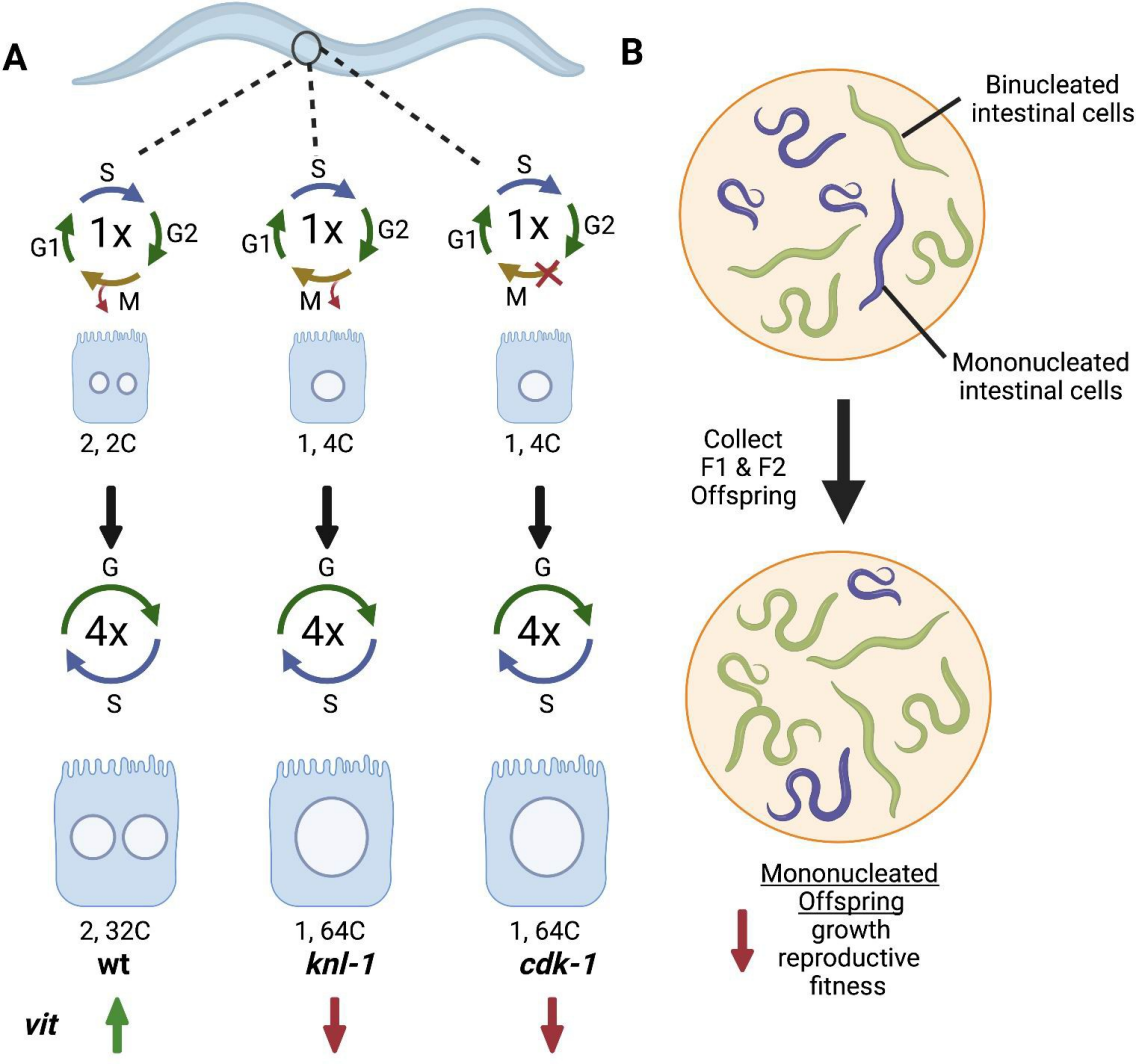
Intestinal binucleation is required for up-regulation of *vit* gene expression and organismal fitness. **(A)** During development, *C*. *elegans* intestinal cells become binucleated through endomitosis and the endocycle. Depletion of KNL-1 inhibits karyokinesis to generate a mononucleated cell, whereas depletion of CDK-1 inhibits entry into M phase. Both mutant conditions generate animals with mononucleated, instead of binucleated, polyploid cells with a total cellular ploidy of 64C. Binucleation is required for optimal *vit* gene expression during intestinal cell development. **(B)** *C*. *elegans* derived from animals with mononucleate intestine have reduced growth and reproductive fitness, while offspring of animals with binucleate intestinal cells more rapidly up-regulated *vit* genes, which are necessary for lipid transport to the oocyte. Created with BioRender.com. *vit*, vitellogenin; wt, wild-type.

In this issue of *PLOS Biology*, van Rijnberk and colleagues [3] utilize the *Caenorhabditis elegans* intestinal model to address this long-standing question. *C*. *elegans* intestinal cells normally arise by incomplete cell cycles known as endoreplication [1,2]. During larval development, the intestinal cells first undergo endomitosis, which truncates M phase prior to cell division (failed cytokinesis), generating a binucleated cell (Fig 1A). The intestinal cell then proceeds through 4 endocycles, cycling between G and S phases without an intervening M phase, doubling the nuclear genome content (C-value) and reaching a total cellular ploidy of 64C with 2, 32C nuclei (Fig 1A).

To address the biological significance of binucleation, van Rijnberk and colleagues [3] conditionally depleted mitotic regulators (KNL-1 and CDK-1) to generate mononucleated intestinal cells. Loss of KNL-1, a kinetochore protein necessary for chromosome segregation, leads to failed karyokinesis, whereas depletion of CDK-1, a regulator of mitotic entry, results in the conversion of the endomitotic cycle to an endocycle (Fig 1A). As a result, the intestinal cells of these animals were composed of 64C mononucleated, instead of binucleated polyploid cells. The change in nucleation, but not total cellular ploidy, yielded animals with equivalent sizes at the cellular, organ, and whole animal level. Taking advantage of this differential genome configuration, the authors then asked how the animal’s intestinal cell ploidy state influences cellular gene expression and organismal fitness (Fig 1A and 1B).

Single-worm RNA sequencing (RNA-seq) identified a handful of differentially expressed transcripts in worms with mononucleated versus binucleated intestinal cells. Among the genes were regulators of fat content including a vitellogenin gene, *vit-2*, which functions to mobilize lipids for transport to the developing oocytes [4]. Using several methods, the author confirmed that *vit-2* as well as other vitellogenin genes were significantly reduced in the mononucleated intestinal cells. However, the reduction of *vit-*2 expression was time dependent and only occurred prior to the transition to the adult stage of development. The authors remarkably discovered that this timed reduction in *vit-2* gene expression had lasting impacts on the worm’s physiology. They found that intestinal binucleation was required for optimal lipid transport to the oocyte, which, in turn, impacted *C*. *elegans* growth and reproductive fitness (Fig 1B). This effect was abrogated indirectly by restoring vitellogenin gene expression via overexpression of 2 global vitellogenin transcriptional regulators (CEH-60 and UNC-62). Therefore, the induction of vitellogenin expression seems to be sufficient to rescue animals with mononucleated intestinal cells, restore normal lipid transport, and overall animal fitness.

It is well established that polyploidy occurs as part of a cell’s normal differentiation program and therefore alters gene expression [1,2]. Such is the case for mammary gland cells in which polyploidization (via binucleation) is necessary for induction of milk production [5]. However, the study by van Rijnberk and colleagues is unique in that it compares gene expression changes between organisms with same total cellular ploidy (64C) that differ only in their ploidy state (i.e., mononucleated versus binucleated). With the emergence of single-cell and nuclear sequencing, several studies have deciphered the gene expression program of polyploid cell types [6–8]. However, only one study to date has directly compared gene expression based on nucleation. Single-cell reverse transcription PCR (RT-PCR) revealed unique gene signatures associated with mononucleated and binucleated tetraploid hepatocytes in the rat liver that correlated with their liver zonation [7]. Here, van Rijnberk and colleagues’ results suggest that binucleation is necessary to ramp up gene expression at a specific time point. If a similar pattern holds true for other organisms, then a subtle change in gene expression would be missed without a complete temporal record of the cell’s gene expression program.

The findings by van Rijnberk and colleagues are also likely to go beyond *C*. *elegans* as binucleated cell types have been identified in many organisms. Superficial cells in the mouse urothelium undergo a similar developmental program to *C*. *elegans* intestinal cells. A binucleated cell is first generated by endomitosis followed by an endocycle to generate an 8C polyploid superficial cell with 2, 4C nuclei [9]. Single-cell RNA-seq has identified cell specific gene expression signatures in the urothelium, but has not investigated how binucleation specifically affects superficial cell gene expression [10].

It has been an enigma as to why a cell adopts a particular ploidy with a single or multiple nuclei per cell. van Rijnberk and colleagues study offers the first clue that ploidy state through the partitioning of the intestinal genome into 2 nuclei has a critical functional and physiological impacts on the cellular and organismal biology. The question now lies in whether a shift from mononucleated to binucleation or even multinucleation would cause a conserved effect on other polyploid cell types. Stress is also known to be an inducer of polyploidization as altered ploidy states are observed in response to injury, aging, and disease in the fruit fly, zebrafish, and mammalian tissues [1,8]. Future studies harnessing the combined power of genomics, cell biological, and genetic tools will help to elucidate the precise impact of ploidy state on the cell, tissue, and organismal biology.
